# Detecting Rare Triple Heteroplasmic Substitutions in the Mitochondrial DNA Control Region: A Potential Concern for Forensic DNA Studies

**Published:** 2011-08-24

**Authors:** Saeid Morovvati, Ziba Morovvati, Reza Ranjbar

**Affiliations:** 1. Research Center for Human Genetics, Baqiyatallah University of Medical Sciences, Tehran, Iran; 2. Research Center of Molecular Biology, Baqiyatallah University of Medical Sciences,Tehran, Iran

**Keywords:** mtDNA, Hypervariable Region, Identification, Forensic Genetics

## Abstract

**Objective:**

Mitochondrial DNA (mtDNA) is a useful tool for population studies, identification of humans and forensic DNA studies. The existence of several hundreds copies of mtDNA per cell permit its extraction from minute or degraded samples. In addition, the level of polymorphism in the hypervariable (HV) region is high enough to permit its use in human identity testing. However, the presence of several heteroplasmy might lead to ambiguous results.

**Materials and Methods:**

This study was an experiental study. This study evaluated heteroplasmy in the HV region of mtDNA in blood samples of 30 Iranians who belonged to ten unrelated families from three sequential generations (grandmother, mother and daughter).

**Results:**

There were no heteroplasmic substitutions in the HV1 region, but analysis of HV2 showed heteroplasmic substitutions in two out ten families. In the first family the grandmother showed heteroplasmy (T/C) in nucleotide positions 146 and 151, however it was not detected in the mother and daughter. In second family, a triple heteroplasmy (T/C) was detected in the daughter in nucleotide positions 146, 151 and 295, but these heteroplasmic substitutions were not obvious in the grandmother and mother.

**Conclusion:**

Heteroplasmy in mtDNA is not a rare phenomenon and probably exists in everyone, but a triple heteroplasmy in one family member is a novel finding. Our results demonstrate that one or two sequence differences between samples in mtDNA do not warrant exclusion. In our study, the average nucleotide difference between unrelated persons in the HV2 region was 2.8 nucleotides, whereas there was a triple heteroplasmy in one person which was not obvious in her family.

## introduction

Human mitochondrial DNA (mtDNA) has proven to be a useful tool for population studies, evolutionary researches and forensic genetics (1). Because of its high copy number, maternal inheritance and high degree of sequence variability between individuals, mtDNA analysis is currently in use by specialized laboratories for identifying the remains of missing persons. In addition, this method of analysis has been proposed for the identification of mass disaster remains, which often consist of a variety of small tissue samples from many individuals. It has several advantages for human identification. The existence of several copies per cell permits mtDNA extraction from minute or degraded samples (2). In addition, the level of polymorphism in the hypervariable (HV) region is high enough to permit its use as an important tool in human identity testing (3,4).

While these features of mtDNA make it a particularly useful target for forensic analyses, there are biological aspects of the organelle that need to be considered to ensure mtDNA typing results are interpreted appropriately. The presence of many hundreds of copies of mtDNA per cell together with its high mutation rate creates the potential for widespread heteroplasmy. Heteroplasmy is defined as appearance of one position with two nucleotide bases in an otherwise unmixed sequence (5). The presence of more than one mtDNA sequence within an individual (heteroplasmy) might lead to ambiguous results in human identification (6).

In this study, we evaluated heteroplasmy in mtDNA from blood samples of 30 Iranians, who belonged to ten unrelated families.

## Materials and Methods

This study was an experimental study. We randomly selected ten volunteer, unrelated Iranian families from three sequential generations (grandmother, mother and daughter). This study was performed in accordance with the Declaration of Helsinki and subsequent revisions. All family members gave written informed consents. This study has been approved in Ethic Committee of Baqiyatallah University of Medical Sciences.

### MtDNA extraction, amplification and sequencing

There were 30 blood samples provided by obtaining 2 ml whole blood from each person in ethylenediaminetetraacetic acid (EDTA) micro tubes. DNA extraction was performed by the standard phenol-chloroform method followed by spectrophotometric quantification of the DNA concentration prior to polymerase chain reaction (PCR) amplification. Two forward and reverse primers were designed for both HV1 and HV2 regions. The sequences of primers were as follows:

HV1: F1 5'-TTAACTCCACCATTAGCACC-3' and R1 5'-CCTGAAGTAGGAACCAGATG-3'
HV2: F2 5'-GGTCTATCACCCTATTAACCAC-3' and R2 5'-CTGTTAAAAGTGCATACCGCCA-3'

### PCR

The PCR master mix for a 25µl reaction consisted of 2.5 µl 10× buffer, 1 µl dNTP, 1 µl primer R, 1 µl primer F, 0.6 µl MgCl_2_, 0.3 µl Taq polymerase, 16.6 µl dH_2_O and 2 µl DNA sample. PCR was performed for both HV1 and HV2 regions. The reaction conditions were as follows: initial denaturation at 95℃ for 3 minutes followed by 30 cycles of denaturation at 94℃ for 1 minute, primer annealing at 56℃ for 1 minute, an extension step at 72℃ for 1 minute, followed by a final linear extension step at 72℃ for 10 minutes. PCR products consisted of two 547 bp and 422 bp DNA segments for HV1 and HV2 regions, respectively. DNA sequencing for 60 PCR products was performed using BigDye terminator (Applied Biosystems) and the ABI PRISM 377 genetic sequencer.

### Characterization of heteroplasmy

The heteroplasmy itself was clearly visible as at least a 1:4 ratio of the two nucleotides on all strands.

## Results

**Fig 1:**
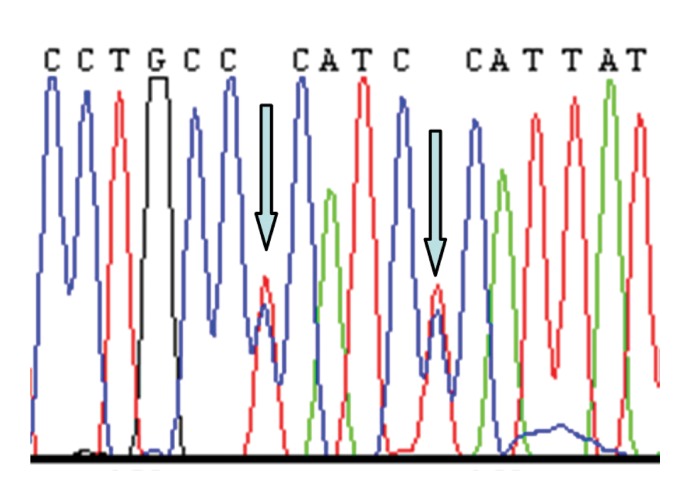
Heteroplasmies T146T/C and C151T/C in mtDNA of grandmother from family 6

**Fig 2:**
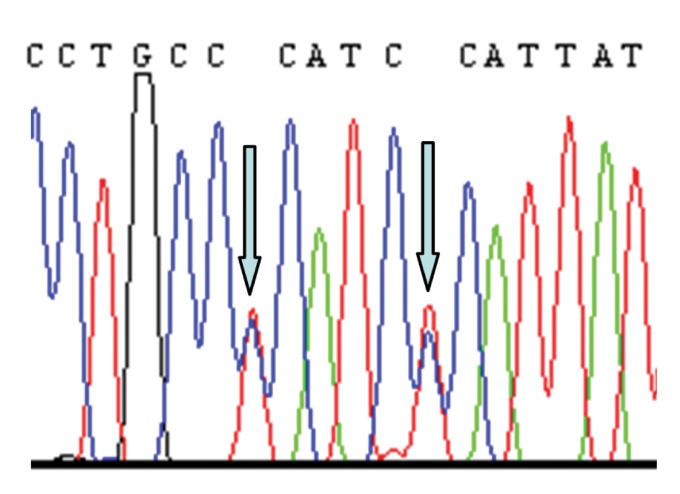
Heteroplasmies T146T/C and C151T/C in mtDNA of daughter from family 8

**Fig 3:**
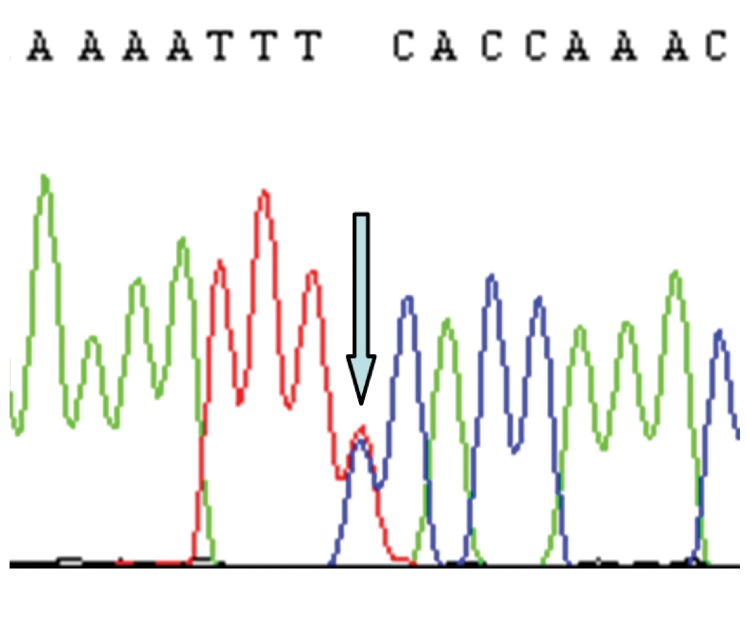
Heteroplasmy C295T/C in mtDNA of the daughter from family 8

The sequence of HV regions were determined and compared with Anderson’s reference (7). There was no heteroplasmy in the HV1 region, but analysis of HV2 showed heteroplasmic substitutions in family numbers 6 and 8. In family number 6 the grandmother showed heteroplasmy (T/C) in nucleotide positions 146 and 151, but in the mother and daughter it was not detected. In family number 8, a triple heteroplasmy was detected in the daughter in nucleotide positions 146, 151 and 295. These heteroplasmies, however, were not obvious in the grandmother and mother (Table 1). Figures 1-3 show electropherograms of the heteroplasmies.

**Table 1:** Heteroplasmic changes in two families compared with Anderson's reference


Family	Positions	Reference	Grandma	Mother	Daughter
6	146	T	T/C	T	T
	151	C	T/C	T	T
8	146	T	C	C	T/C
	151	C	C	C	T/C
	295	C	T	T	T/C


## Discussion

Mitochondrial DNA analysis is a growing area of forensic testing in many countries (8). The aim of this study was to evaluate heteroplasmic phenomenon in the control-region of mtDNA in Iranian families by blood samples. We have detected 5 heteroplasmic sites in 30 persons. To our knowledge, a triple heteroplasmy in one member is a novel finding.

Several studies have questioned whether the differences between mother and offspring may be the consequence of the so-called bottleneck hypothesis. The bottleneck theory has been proposed to explain the results from the examination of a heteroplasmic site in four generations of Holstein cows (9). The results are similar to our results in the present study on human mtDNA, namely: i) the proportions of heteroplasmy could change in a single generation, ii) the offspring of a woman could have different genotypes, and iii) the heteroplasmy could revert to homoplasmy in only two to three generations. This bottleneck theory suggests that mtDNA from a few mitochondria is selectively amplified during oogenesis or development and thus, a mutant genotype can become predominant and fixed in future generations.

Other laboratories have also found that heteroplasmies are not always inherited in the same proportions between generations or between tissues of the same individual (10-12). In earlier studies, mtDNA heteroplasmy was believed to be a rare phenomenon in normal populations (13). However, more recently, there have been a number of reports of the detection of heteroplasmies in the non-coding region (14,15).

## Conclusion

Therefore, heteroplasmy in human mtDNA is not a rare phenomenon and probably exists in everyone. Our results demonstrate that one or two sequence differences between samples in the mtDNA do not warrant exclusion in an identification test. HV2 cannot solely be used in forensic research because in our study the average nucleotide difference between unrelated persons was 2.7 nucleotides. We also found a triple heteroplasmy.

At this time, more analysis is needed before any consensus should be reached as to the number of nucleotide differences required for exclusion. Heteroplasmic evaluation in sequential generations within larger populations in future research can reveal the role and importance of heteroplasmy in forensic DNA studies.
